# Mortality and Causes of Death among Older Inpatients with Non-Severe Coronavirus Disease 2019 during the Omicron Era: A Retrospective Cohort Study in a Community Hospital

**DOI:** 10.31662/jmaj.2025-0465

**Published:** 2026-05-08

**Authors:** Maine Takemura, Masahisa Arahata, Masato Kuriyama

**Affiliations:** 1 Department of General Medicine, Nanto Municipal Hospital, Nanto, Japan; 2 Nanto Community Medical Support Unit, Toyama University Hospital, Toyama, Japan; 3 Department of Internal Medicine, Nanto Municipal Hospital, Nanto, Japan

**Keywords:** COVID-19, SARS-CoV-2, Omicron variant, cause of death, comorbidity, COVID-19-related death

## Abstract

**Introduction:**

Quantifying the impact of coronavirus disease (COVID) 2019 (COVID-19) on mortality has become increasingly challenging in the Omicron-variant era. This difficulty arises from reduced excess mortality, the presence of long COVID, and the lack of a strict definition of COVID-19-related death, which has led to inconsistencies in reported mortality rates. Accurate cause-of-death statistics are essential for health care policymaking.

**Methods:**

We conducted a retrospective cohort study in patients aged ≥65 years who were admitted to the COVID-19 isolation ward of our hospital between July 1, 2021 and September 30, 2023. The primary outcome was survival to hospital discharge. Causes of death were classified according to the World Health Organization definition into four categories: COVID-19, COVID-19-associated complications, pre-COVID-19 medical conditions, and post-COVID-19 complications. The latter two categories were considered deaths unrelated to COVID-19.

**Results:**

A total of 182 patients with mild or moderate COVID-19 were included for analysis. Among these, 23 (13%) died during hospitalization. Those who died were more likely to have moderate COVID-19, impaired physical or eating functions, and multiple comorbidities than were survivors. Of the 23 deaths, only 8 (35%) were attributed to COVID-19 or COVID-19-associated complications, whereas 15 (65%) were due to pre-COVID-19 medical conditions or post-COVID-19 complications. Concordance between Japanese public criteria for COVID-19-related death and our classification method was low (Cohen’s κ = 0.223).

**Conclusions:**

In this cohort of older patients with mild or moderate COVID-19, in-hospital mortality was relatively high. However, most deaths were not directly attributable to COVID-19. Larger studies are needed to further clarify mortality outcomes in this population.

## Introduction

Coronavirus disease (COVID) 2019 (COVID-19) was declared a global pandemic by the World Health Organization (WHO) on March 11, 2020 ^(1)^. It caused an enormous number of deaths and a 2.2-year decrease in life expectancy worldwide in the first 3 years ^(2)^. Since then, COVID-19 mortality has decreased owing to the widespread use of vaccines and the dominance of the Omicron variant. However, the pandemic is not over, and COVID-19 continues to affect mortality rates ^(1), (3), (4)^. In addition, the post-viral syndrome, long COVID, has become a major public health problem ^(5), (6)^.

There have been many attempts to grasp the causes of death in patients with COVID-19. However, accurate outcomes are difficult to clarify in the Omicron-variant era. There are several reasons for this. First, excess mortality was previously considered an important means of estimating mortality rates directly and indirectly attributable to COVID-19 ^(7)^. However, this had already begun to decrease by 2023 in many countries ^(8), (9)^. Excess mortality has also been found inconsistent between diseases and countries ^(7), (9)^, and the underlying mortality data usually underestimate the number of deaths compared with data from death certificates, health care billing records, or medical chart reviews ^(10), (11), (12)^. Second, it is unclear whether the risk factor for mortality in cases of the Omicron variant is the same as that in cases of the other variants. Moreover, the clinical course of COVID-19 has been altered by widespread vaccination and new antivirals ^(13)^. Recently, frailty was reported as one of the greatest factors associated with clinical outcomes in older patients with COVID-19 even in an era when coronavirus vaccines were widespread and the Omicron variant was dominant ^(14), (15), (16)^. However, causes of death were not strictly evaluated in those studies. Third, long COVID makes it difficult to pinpoint the origin of symptoms ^(17)^. Suboptimal recognition and responses to long COVID have been reported among physicians ^(18)^. Although long COVID may be a mortality risk, the situation is not fully understood ^(19), (20), (21)^. As a result, the true causes of death in patients with COVID-19 are not always clear. Cause-of-death statistics are indispensable for health care policymaking, and ensuring their accuracy is imperative.

In Japan, COVID-19 was described as “the Novel Influenza etc.” by the Infectious Diseases Control Law on February 13, 2020, and legal notifiable surveillance for COVID-19 cases was initiated ^(22)^. The Omicron variant emerged in December 2021, and COVID-19 mortality began to decrease in early 2022 ^(23)^. However, these mortality rates were not derived from the national surveillance system (Health Center Real-Time Information-Sharing System on COVID-19 [HER-SYS]) ^(24)^, and no strict definition of COVID-19-related death (CRD) was applied ^(25)^. It has also been reported that the HER-SYS does not collect all COVID-19 outcome data ^(25)^. Moreover, CRD adopted by the Japanese Ministry of Health, Labour, and Welfare (MHLW) is not identical to the “definition for deaths due to COVID-19” according to WHO ^(26), (27)^. After the epidemic was brought under control, COVID-19 was reclassified as a category V infectious disease on May 8, 2023, and notifiable surveillance transitioned to sentinel surveillance ^(22)^. Consequently, national surveillance has failed to provide accurate data on COVID-19 mortality, and real-time monitoring is no longer possible. Under these circumstances, it is worth noting that CRDs during the Omicron epidemic were concentrated among older adults ^(28)^.

In this observational study, we aim to obtain accurate mortality data and identify the true causes of death especially in older patients with COVID-19 who required hospitalization.

## Materials and Methods

### Study design

This single-center retrospective cohort study investigated mortality and cause of death in older inpatients with COVID-19. The study was approved by the institutional review board of Nanto Municipal Hospital (approval number shiminbyouin-932). Because the study was non-interventional, the informed consent requirement was waived per the Ethical Guidelines for Medical and Biological Research Involving Human Subjects ^(29)^. This report follows the Strengthening the Reporting of Observational Studies in Epidemiology guidelines for observational studies ^(30)^.

### Setting and participants

**Figure 1.**
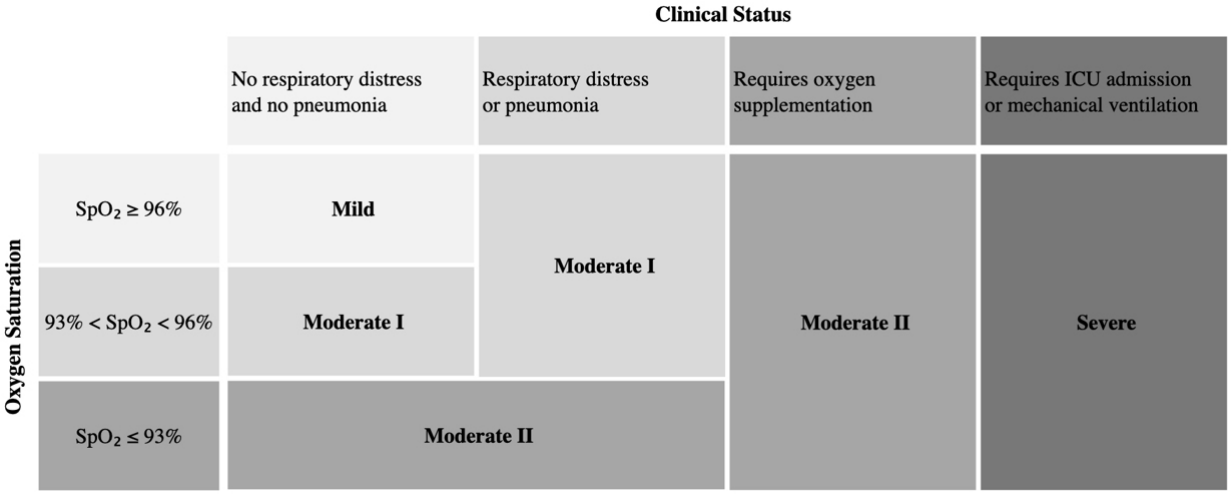
Severity categories based on the MHLW criteria. The figure was originally created by the authors, but the severity criteria follow *the Guidelines* for the Clinical Management of COVID-19 ^(31)^. ICU: intensive care unit; MHLW: Ministry of Health, Labour, and Welfare; SpO2: saturation of peripheral oxygen.

Participants hospitalized with COVID-19 between July 1, 2021 and September 30, 2023 at Nanto Municipal public hospital in the Toyama prefecture of Japan were identified from our institutional medical records. The inclusion criteria were (1) COVID-19 diagnosed by a severe acute respiratory syndrome coronavirus 2 (SARS-CoV-2) antigen or polymerase chain reaction test; (2) being admitted to a COVID-19 isolation ward for at least one day; (3) aged ≥65 years. Patients admitted after the isolation period and those with false-positive test results for SARS-CoV-2 were excluded. This is because COVID-19 was not associated with the necessity for hospitalization in these patients. In medical practice, the criteria for isolating patients with COVID-19 were consistent with the Infectious Diseases Control Law, which stipulated that patients with COVID-19 should be isolated for 10 days after symptom onset. Patients with severe COVID-19, as defined by the MHLW criteria (Figure 1) ^(31)^, were also excluded from the analysis because our hospital did not have an intensive care unit and sufficient capacity to treat them. They were transferred to advanced-care hospitals as necessary.

### Variables and measurements

The primary outcome was survival to discharge from the hospital. Other data included in our analysis were baseline characteristics (age, severity of COVID-19, pre-COVID-19 medical conditions, pre-admission medications, medical history, physical functions, cognitive functions, nutritional status, the Quick Sequential Organ Failure Assessment [qSOFA] score, laboratory data, and X-ray imaging data), interventions during hospitalization (treatment, rehabilitation, diet, and artificial hydration and/or nutrition [AHN]), and secondary outcomes (cause of death, length of hospitalization, and post-COVID-19 complications). The severity of COVID-19 was assessed at the time of diagnosis by the attending physicians in accordance with the criteria provided by MHLW (Figure 1) ^(31)^. Physical functions were evaluated using the Bedriddenness Rank (BR) and Functional Independence Measure scales ^(32), (33)^. Cognitive functions were evaluated using the Cognitive Function Score and the Hasegawa Dementia Scale-Revised ^(32), (34)^. Eating and swallowing functions were evaluated with the Functional Oral Intake Scale (FOIS) ^(35)^, the Food Intake Level Scale (FILS) ^(36)^, and the need for AHN.

Pre-COVID-19 medical conditions were defined as (1) acute illness identified before COVID-19 onset or those that coexisted at COVID-19 diagnosis; and (2) illness requiring inpatient treatment. Post-COVID-19 complications were defined as (1) acute illness that occurred after COVID-19 onset; and (2) illness requiring or prolonged hospitalization. Information on causes of death was obtained from death certificates, which were written by the attending physicians. Causes of death were classified into four categories: COVID-19, COVID-19-associated complications (onset during treatment for COVID-19 or apparently related to COVID-19), pre-COVID-19 medical conditions, and post-COVID-19 complications (onset after recovery from COVID-19 and clearly unrelated to COVID-19). Deaths attributed to COVID-19 and COVID-19-associated complications were defined as those resulting directly and indirectly from COVID-19, respectively. The other two categories were considered unrelated to COVID-19. Cause of death categorization was performed by two researchers after an independent review of the medical records, and any disagreements were resolved through discussion. The classification methods were based on the WHO guidelines ^(27)^. Notably, the guidelines suggest that conditions contributing to death described in part 2 of Frame A in a death certificate (not a part of direct chains to the death) should not be regarded as COVID-19 death. However, we allowed conditions to be classified as COVID-19-associated complications even when COVID-19 was described in panel 2 (Japanese format: “Column II”) of the death certificate. Because there is no definitive criterion for distinguishing whether a condition is related to COVID-19, we adopted a broader interpretation and conducted discussions among multiple researchers until a consensus was reached.

### Statistical analysis

Given this was an observational study, we did not calculate the sample size required for sufficient power. However, all consecutive patients from the study period who met the study criteria were recruited. The mortality rate was defined as the proportion of the cohort who died during hospitalization. This was expressed as a percentage (%) and 95% confidence interval (CI). Participants were divided into a death group and a survival group for comparison of variables. Normally distributed continuous variables were expressed as mean (range) and compared using t tests. Continuous variables with skewed distributions were expressed as median (interquartile range [IQR]) and compared using the Mann-Whitney U test. Fisher’s exact test was used when comparing nominal and ordinal variables. Participants with missing data were included in the statistical analyses, but the number of patients with data was represented in the results (as presented in the tables). Multivariable analysis was planned to exclude confounding factors but could not be performed owing to insufficient sample size. The causes of death in the death group were determined using the Japanese public criteria for CRD published by MHLW (the MHLW criteria) ^(26)^, and the definitions previously given in the variables and measurements subsection. Consistency between the two categorical datasets on causes of death judged by these two methods was assessed using Cohen’s kappa. All statistical analyses were performed using the EZR (Saitama Medical Center, Jichi Medical University, Saitama, Japan) graphical user interface for R (The R Foundation for Statistical Computing, Vienna, Austria) ^(37)^. p-Values < 0.05 were considered statistically significant.

## Results

**Figure 2.**
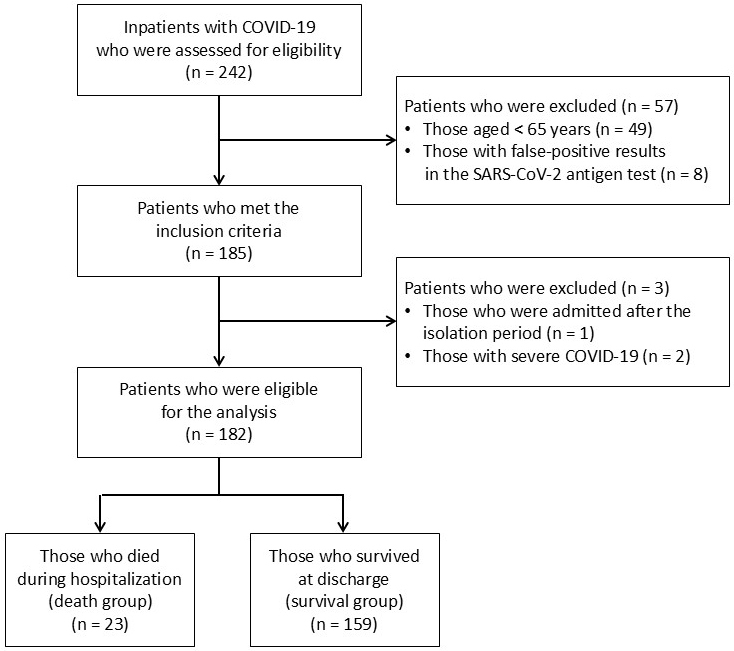
Flow diagram of the patient selection and recruitment process. Eligible participants were identified and recruited according to the inclusion and exclusion criteria of this study. COVID-19: coronavirus disease 2019; SARS-CoV-2: severe acute respiratory syndrome coronavirus 2.

During the study period, 242 patients with COVID-19 were admitted to our hospital. Notably, during the first 6 months of the period, all inpatients with COVID-19 were younger than 65 years, and the first COVID-19 patient aged ≥65 years was hospitalized on January 31, 2022, when the Omicron variant had already been dominant in Japan ^(25), (38)^. Among these, 182 (99 men and 83 women) met our study criteria (Figure 2). The median age of the eligible participants was 87 years (IQR 79-92). A total of 23 deaths occurred in the cohort during hospitalization. The mortality rate was calculated as 13% (95% CI: 8-18) (Figure 2).

The baseline characteristics of the death and survival groups are listed in Table 1. The death group had significantly higher proportions of COVID-19 of moderate (rather than mild) severity (p < 0.01), qSOFA scores ≥2 (p = 0.01), severe BR (p = 0.045), low eating and swallowing functions (FOIS level ≤3 [p = 0.03] and/or FILS level ≤6 [p = 0.03]), pre-COVID-19 medical conditions (mainly heart failure) (p = 0.01), and patients prescribed oral steroids than did the survival group (p = 0.04) (Table 1). The laboratory data of the death group included significantly lower platelet count, serum total protein, and serum albumin levels, and significantly higher serum C-reactive protein and serum urea nitrogen levels than in the survival group (Table 1). There was also a significant difference in D-dimer levels, but this was invalid owing to insufficient data; the missingness rates were 65% (15/23) and 67% (107/159) in the death and survival group, respectively (Table 1). Although the SARS-CoV-2 genotype is not identified in our hospital, it is likely that most participants had the Omicron variant because this accounted for most SARS-CoV-2 genotypes in Japan from January 2022 to the end of the study period ^(25), (38)^.

**Table 1.** Baseline Characteristics of Study Participants.

Variables	Death group (n = 23)	Survival group (n = 159)	p
Age (years), median (IQR)	87 (82-94)	86 (79-92)	0.39
Sex―male, n (%)	17 (74)	82 (52)	0.07
Severity of COVID-19, n (%)			<0.01
Mild	1 (4)	51 (32)	
Moderate I	4 (17)	35 (22)	
Moderate II	18 (78)	73 (46)	
qSOFA ≥2, n (%)	10 (43)	28 (18)	0.01
High risk for severe COVID-19,* n (%)	23 (100)	147 (92)	0.37
Vaccination, n (%)			0.37
One or more	13 (57)	111 (70)	
None	2 (9)	9 (6)	
Unknown	8 (35)	39 (25)	
Residence at the onset of COVID-19, n (%)			0.56
Own home	12 (52)	94 (59)	
Nursing home	2 (9)	21 (13)	
Hospitalized**	9 (39)	44 (28)	
Acute care ward	8	37	
Community-based integrated care ward	1	5	
Rehabilitation ward	0	2	
CCI score, median (IQR)	7 (6-8)	7 (6-8)	0.18
≥6, n (%)	21 (91)	136 (86)	0.75
≥7, n (%)	16 (70)	91 (57)	0.37
≥8, n (%)	9 (39)	44 (28)	0.33
Physical functions			
Bedriddenness rank, n (%)			0.045
Independent	1 (4)	27 (17)	
J or A	5 (22)	59 (37)	
B or C	17 (74)	73 (46)	
FIM score, median (IQR)	28 (18-36)	33 (24-49)	0.14
No. of patients with data	12	88	
Cognitive functions			
Cognitive function score, n (%)			0.18
Independent	2 (9)	38 (24)	
I or II	7 (30)	51 (32)	
III, IV, or M	14 (61)	38 (44)	
HDS-R score, median (IQR)	7 (6-14)	6 (0-16)	0.52
No. of patients with data	6	31	
Eating and swallowing functions			
FOIS level ≤3, n (%)	21 (91)	107 (67)	0.03
FILS level ≤6, n (%)	21 (91)	110 (69)	0.03
Dependent on an AHN, n (%)	1 (4)	4 (3)	0.50
Pre-COVID-19 medical conditions, n (%)	19 (83)	87 (55)	0.01
Bacterial infection	12 (52)	48 (30)	0.06
Malignant tumor	2 (9)	8 (5)	0.62
Bone fracture	0 (0)	9 (6)	0.61
Heart failure	3 (13)	2 (1)	0.02
Interstitial pneumonia	0 (0)	4 (3)	1.00
Bronchial asthma/COPD	1 (4)	2 (1)	0.34
Eating problems	1 (4)	4 (3)	0.50
Thrombosis/embolism	0 (0)	5 (3)	1.00
Major bleeding	0 (0)	3 (2)	1.00
Other	2 (9)	7 (4)	0.32
Two or more of above diseases	2 (9)	5 (3)	0.22
Regular medication (pre-dating COVID-19 onset), n (%)			
Antiplatelets	6 (25)	44 (28)	1.00
Anticoagulants	3 (13)	16 (10)	0.72
Ca blockers	7 (29)	52 (33)	0.82
ACE inhibitors or ARBs	10 (42)	53 (33)	0.49
β blockers	8 (33)	27 (17)	0.09
Diuretics	8 (33)	38 (24)	0.32
Hypoglycemia drugs	4 (17)	36 (23)	0.61
Insulin	1 (4)	3 (2)	0.43
Statins	3 (13)	37 (23)	0.30
Stomach medicine	9 (38)	67 (42)	0.83
Antidementia drugs	5 (21)	33 (21)	1.00
Antipsychotics	3 (13)	16 (10)	0.72
Trace element preparations	6 (25)	25 (16)	0.25
Laxatives	9 (38)	66 (41)	0.83
Expectorants	4 (17)	27 (17)	1.00
Steroids	5 (21)	11 (7)	0.04
Immunosuppressants	1 (4)	4 (3)	0.51
Chemotherapy	0 (0)	5 (3)	1.00
No. of medication types pre-admission, median (IQR)	6 (3-9)	5 (4-7)	0.39
Laboratory data at diagnosis, all median (IQR)			
WBC (μL)	6640 (4940-9705)	6360 (4690-8410)	0.50
No. of patients with data	23	149	
Neutrophils (μL)	5550 (3600-7700)	4895 (3150-6355)	0.26
No. of patients with data	21	142	
Lymphocytes (μL)	890 (570-1140)	910 (653-1258)	0.38
No. of patients with data	21	142	
Hemoglobin (g/dL)	11.7 (10.4-13.4)	11.3 (9.9-12.6)	0.32
No. of patients with data	23	149	
Platelet count (×10^3^/μL)	135 (115-160)	185 (142-230)	<0.001
No. of patients with data	23	149	
Serum CRP (mg/dL)	4.8 (2.1-10.4)	2.3 (1.1-5.3)	0.02
No. of patients with data	23	146	
Serum total protein (g/dL)	6.1 (5.5-6.5)	6.5 (6.1-7.0)	0.005
No. of patients with data	21	137	
Serum albumin (g/dL)	3.2 (2.3-3.4)	3.4 (3.0-3.8)	0.005
No. of patients with data	19	130	
Serum UN (mg/dL)	22.5 (13.6-51.4)	17.0 (12.4-23.7)	0.04
No. of patients with data	23	149	
Serum creatinine (mg/dL)	0.81 (0.61-1.40)	0.84 (0.64-1.13)	0.91
No. of patients with data	23	149	
D-dimer (μg/mL)	1.86 (1.28-3.26)	0.89 (0.48-1.55)	0.03
No. of patients with data	8	52	
Chest X-ray performed, n (%)	4 (17)	29 (18)	1.00
Pneumonia indicated by X-ray, n (%)	3 (75)	6 (21)	0.052
Chest CT performed, n (%)	22 (96)	141 (89)	0.48
Pneumonia indicated by CT, n (%)	16 (73)	61 (43)	0.01
Imaging not performed, n (%)	1 (4)	13 (8)	1.00

*High risk for severe COVID-19 was defined as having at least one of the following factors: (1) age ≥80 years, (2) the presence of an uncontrolled underlying disease, or (3) two or more comorbidities associated with severe COVID-19, as specified in *the Guidelines for the Clinical Management of COVID-19*, Version 10.1 ^(31)^. The definition was based on Table 4-3 of the same guideline ^(31)^. **All 53 patients in whom COVID-19 developed during hospitalization were admitted to our hospital at the time of onset. The numbers shown for each ward indicate the distribution of these hospitalized cases. No significant difference in ward distribution was observed between the two groups (p = 1.00). ACE: angiotensin converting enzyme; AHN: artificial hydration and nutrition; ARB: angiotensin II receptor blocker; Ca: calcium; CCI: Charlson Comorbidity Index; COPD: chronic obstructive pulmonary disease; COVID-19: coronavirus disease 2019; CRP: C-reactive protein; CT: computed tomography; FILS: food intake level scale; FIM: functional independence measure; FOIS: functional oral intake scale; HDS-R: Hasegawa dementia scale-revised; IQR: interquartile range; qSOFA: quick sequential organ failure assessment; UN: urea nitrogen; WBC: white blood cell.

Patient treatments and interventions during hospitalization are presented in Table 2. The proportion of patients who had received any treatment for COVID-19 was significantly lower in the death group (74% vs 92%, p = 0.02). The main reason for non-treatment was identified as exceeding the optimal treatment period; the patients who were admitted to the hospital 7 days after onset were 3 of 6 patients in the death group and 8 of 13 patients in the survival group. However, in the remaining patients, definitive reasons could not be determined, given multiple factors may have been involved, including attending physician preference, patient consent, organ dysfunction, comorbidities, and drug availability. The frequency of fasting instructions on admission and AHN requirement after COVID-19 treatment were significantly higher in the death group (Table 2).

**Table 2.** Treatments and Interventions Received by Older Inpatients with Non-Severe COVID-19.

Variables	Death group (n = 23)	Survival group (n = 159)	p
Medications for COVID-19, n (%)			
None	6 (26)	13 (8)	0.02
Antivirals	16 (70)	137 (86)	0.06
Antibodies	1 (4)	10 (6)	1.00
Immunosuppressants	3 (13)	9 (6)	0.18
Dependent on AHN after the previously mentioned COVID-19 treatment,* n (%)	18 (86)	55 (35)	<0.001
Rehabilitation**			
PT intervention (units), median (IQR)	0 (0-5)	2 (0-13)	0.12
OT intervention (units), median (IQR)	0 (0-9)	0 (0-12)	0.16
ST intervention (units), median (IQR)	0 (0-1)	0 (0-0.5)	0.92
No intervention, n (%)	13 (44)	75 (53)	0.50
Duration between COVID-19 onset and the start of rehabilitation (days), median (IQR)	12 (10-13) (n = 10)	12 (7-14) (n = 84)	0.73
Fasting instructions on admission, n (%)	17 (74)	45 (28)	<0.001
Duration of fasting within 30 days of admission (days), median (IQR)	2 (1-16)	0 (0-1)	<0.001

*The percentage for this variable was calculated among the patients who survived after COVID-19 treatment. AHN included continuous infusions (peripheral and/or central venous) and tube feeding. **One unit = 20 minutes of rehabilitation. AHN: artificial hydration and nutrition; COVID-19: coronavirus disease 2019; IQR: interquartile range; OT: occupational therapist; PT: physical therapist; ST: speech-language-hearing therapist.

**Figure 3.**
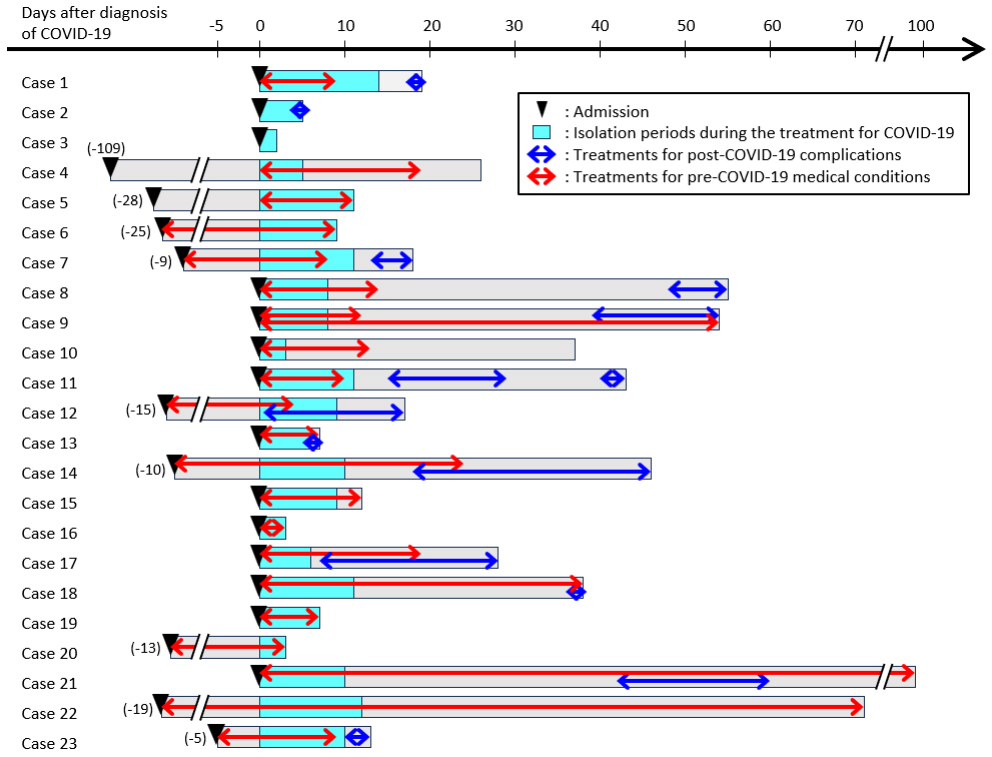
Clinical course of the patients who died during hospitalization. The date of COVID-19 diagnosis was set at day 0 of the horizontal axis in each patient. In some cases, because the day of onset differed from the day of diagnosis, the isolation period was <10 days. The right edge of each bar indicates the timing of death. COVID-19: coronavirus disease 2019.

Of the 23 deaths, only eight (35%) were attributed to COVID-19 or COVID-19-associated complications (Table 3), whereas 15 (65%) were due to pre-COVID-19 medical conditions or post-COVID-19 complications. Post-COVID-19 complications occurred much more frequently in the death group (Table 3). The clinical information that informed these judgments is presented in Table 4 and Figure 3. In the death group, COVID-19 developed in nine patients (39%) after admission, and these patients died during treatments for comorbidities and complications (Figure 3). Only two patients died during short-term care for COVID-19 (Table 3); they were not transferred to advanced-care hospitals because they were considered unlikely to benefit from intensive treatment owing to severe comorbidities. As a result, the most frequent cause of death was exacerbation of pre-COVID-19 medical conditions (eight patients), followed by post-COVID-19 complications (seven patients) (Table 4). Nine of the deaths classified as CRD by the MHLW criteria were determined to be unrelated to COVID-19 (pre-COVID-19 medical conditions or post-COVID-19 complications) according to our study method (Table 4). In the death certificates of these nine patients, COVID-19 was recorded in column II, which denotes conditions not directly associated with death, as confirmed by a review of their clinical course. The Cohen’s kappa coefficient showed low consistency between the cause of death classification methods (the MHLW criteria and our classification method, which are presented in Table 4) (0.22 [95% CI: −0.14 to 0.59]) (Table 5).

**Table 3.** Outcomes in Older Hospital Inpatients with Non-Severe COVID-19.

Variables	Death group (n = 23)	Survival group (n = 159)	p
Death directly or indirectly associated with COVID-19, n (%)	8 (35)	0	<0.001
Death during short-term care for COVID-19, n (%)	2 (9)	0	0.02
Post-COVID-19 complications,* n (%)	17 (81)	31 (20)	<0.001
Bacterial infections, n (%)	10 (48)	21 (13)	<0.001
Heart failure, n (%)	2 (10)	0	0.01
Interstitial pneumonia, n (%)	2 (10)	0	0.01
Bleeding events, n (%)	3 (14)	2 (1)	0.01
Other complications, n (%)	2 (10)	12 (8)	0.67
Duration of isolation (days), median (IQR)	8 (5-10)	10 (7-11)	0.03
Duration of hospitalization (days), median (IQR)			
All patients	32 (14-49)	25 (12-48)	0.83
Patients in whom COVID-19 developed during hospitalization	34 (27-39) (n = 9)	47 (31-69) (n = 44)	0.27

Post-COVID-19 complications were defined as acute illness that occurred after the onset of COVID-19. The illness must be of sufficient severity to require or prolong hospitalization and to necessitate short-term medical care. At least one of the two researchers for this study must consider these criteria to be met. More than one complication occurred in some patients. COVID-19: coronavirus disease 2019; IQR: interquartile range.

**Table 4.** Identification of the Causes of Death among Older Inpatients with Non-Severe COVID-19 Who Died Before Hospital Discharge.

Case no.	Cause of death on death certificate	CRD or CUD*	Days between COVID-19 diagnosis and death	COVID-19 treatment completed before death	Causes of death as judged by this study
1	I) Upper gastrointestinal bleeding and AP II) COVID-19, previous stroke, previous MI, and dementia	CRD	19	Yes	Post-COVID-19 complications
2	I) AAA rupture II) COVID-19, CHF, CKD, and hypertension	CRD	5	No	Post-COVID-19 complications
3	I) Acute subdural hematoma II) COVID-19	CRD	2	Yes	COVID-19
4	I) Acute cholecystitis II) Lumbar compression fracture and frailty syndrome	CUD	25	Yes	Pre-COVID-19 medical conditions
5	I) Prostatitis, UTI, and prostate cancer II) COVID-19 and diabetes	CRD	10	No	COVID-19-associated complications
6	I) CO_2_ narcosis II) CHF	CUD	8	No	Pre-COVID-19 medical conditions
7	I) AP and aftereffects of stroke II) Acute cholecystitis	CUD	17	Yes	Post-COVID-19 complications
8	I) AP and acute cerebellar stroke II) COVID-19	CRD	55	Yes	Post-COVID-19 complications
9	I) AP and dysphagia II) COVID-19, schizophrenia, and previous MI	CRD	54	Yes	Pre-COVID-19 medical conditions
10	I) Bacterial pneumonia II) COVID-19	CRD	37	Yes	Pre-COVID-19 medical conditions
11	I) RF (type I) and AP II) COVID-19, dementia, and dysphagia	CRD	43	Yes	Post-COVID-19 complications
12	I) IP II) None	CUD	16	Yes	COVID-19-associated complications
13	I) ARDS, COVID-19, anti-ARS-Ab syndrome, and IP II) Angina pectoris and previous stroke	CRD	7	No	COVID-19
14	I) Acute CHF, malnutrition, and dysphagia II) None	CUD	46	Yes	Post-COVID-19 complications
15	I) RF (type II), AP, and emphysema II) COVID-19	CRD	12	Yes	COVID-19-associated complications
16	I) COVID-19 II) Past malignant lymphoma and hypoadrenocorticism	CRD	2	No	COVID-19
17	I) AP and frailty syndrome II) COVID-19	CRD	28	Yes	Pre-COVID-19 medical conditions
18	I) Acute CHF, CHF, and aortic valve disease II) COVID-19	CRD	38	Yes	Pre-COVID-19 medical conditions
19	I) AP and cerebral hemorrhage II) COVID-19	CRD	7	No	COVID-19-associated complications
20	I) Pulmonary edema II) COVID-19	CRD	2	Yes	COVID-19
21	I) CHF II) COVID-19 and AP	CRD	98	Yes	Pre-COVID-19 medical conditions
22	I) Prostate cancer II) Dementia	CUD	72	Yes	Pre-COVID-19 medical conditions
23	I) Septic shock and bacterial pneumonia II) None	CUD	14	Yes	Post-COVID-19 complications

*In Japan, a death certificate describes I) the direct cause of death and the related disease(s); and II) factors not directly associated with death but affecting the clinical course. If a term indicating CoV-SARS-2 infection (COVID-19, coronavirus infection, SARS, or other) was used for either I or II, the cause of death was regarded as CRD according to the MHLW criteria ^(26)^. AAA: abdominal aortic aneurysm; AP: aspiration pneumonia; ARDS: acute respiratory distress syndrome; ARS-Ab: aminoacyl-tRNA synthetase antibody; CHF: chronic heart failure; CKD: chronic kidney disease; CO_2_: carbon dioxide; COVID-19: coronavirus disease 2019; CRD: COVID-19-related death; CUD: COVID-19-unrelated death; IP: interstitial pneumonia; MI: myocardial infarction; MHLW: Ministry of Health, Labour, and Welfare; RF: respiratory failure; UTI: urinary tract infection.

**Table 5.** Crosstabulation of Causes of Death Based on the Japanese Public Criteria and the Results of This Study.

Causes of death based on the MHLW criteria	Causes of death based on this study		Total
	COVID-19 or COVID-19-associated complications	Pre-COVID-19 medical conditions or post-COVID-19 complications	
CRD	7	9	16
CUD	1	6	7
Total	8	15	23

The Cohen’s kappa coefficient value was 0.223 (95% CI: −0.139 to 0.585), which indicates a low consistency between the two assessments. CI: confidence interval; COVID-19: coronavirus disease 2019; CRD: COVID-19-related death; CUD: COVID-19-unrelated death; MHLW: Ministry of Health, Labour, and Welfare.

## Discussion

This study investigated mortality among older inpatients with non-severe COVID-19 and the causes of death. Despite all patients having only mild-to-moderate COVID-19, we found a mortality rate of 13% (95% CI: 8-18). The death group had more cases of moderate severity, qSOFA ≥2, a BR of B/C, FOIS level ≤3, FILS level ≤6, coexisting acute illness, and a higher frequency of oral steroid use (Table 1). These characteristics suggest that death during hospitalization was more likely in patients who were frail with decreased eating functions, and fasting instructions may have worsened their outcomes (Table 2). However, the causal relationship between fasting and patient outcomes could not be determined by this analysis because patients who required fasting may have had unfavorable baseline conditions that could contribute to poor prognosis. The instructions for fasting were ordered by the attending physicians based on the patients’ conditions. However, owing to the small sample size, we were unable to perform multivariate analysis to identify the risk factors. The same limitation applies to treatment rates. The proportion of patients who had received any treatment for COVID-19 was significantly lower in the death group (Table 2). However, the treatment rates in this study (90%) were relatively high compared with previous studies in Japan ^(39), (40)^, so it is doubtful whether the non-treatment contributed to the high mortality rate in all patients in this study. The most notable finding was that more than half of the deaths in our cohort were unrelated to COVID-19 (Tables 3 and 4). According to the WHO definition, COVID-19 recorded in column II of a death certificate should not be regarded as a cause of death ^(27)^. In this study, we strictly adhered to the WHO definition, and the significance of COVID-19 in column II was carefully evaluated by reviewing each patient’s clinical course. In some cases, COVID-19 had been incorrectly entered in column II instead of column I.

The mortality rate in this sample appears to be high compared with the population in previous reports ^(4), (13)^. Klompas et al. ^(4)^ found a mortality rate of hospital-onset COVID-19 in the Omicron period of 7.0% (73 of 1037 patients). McNeil et al. ^(41)^ reported a mortality rate among inpatients of 6% (12 of 215 patients) within 30 days of COVID-19 diagnosis. In contrast, the mortality rate of hospital-onset COVID-19 was 17% (9 of 53 patients) in our study (Table 1). These previous studies are not easily comparable with ours owing to differences in inclusion criteria and participant characteristics. For example, the mean age of our participants was substantially higher. Zhong et al. ^(42)^ reported a mortality rate of 22.5% (68 of 302) among patients aged 65 years and older with community-acquired COVID-19, which was significantly higher than that observed in our study (p < 0.01, Fisher’s exact test). This discrepancy may be attributable to differences in disease severity, given their cohort included 33.1% of severe COVID-19 cases, whereas our study excluded such patients. Our study included participants with both hospital-onset and community-acquired COVID-19, indicating the mortality was relatively high regardless of primary admission reason (11% and 17%, respectively). Therefore, our findings suggest that hospitalized older patients with COVID-19 are at risk of death regardless of the reason for their admission.

We found that the main causes of death among inpatients with COVID-19 were comorbidities and complications judged to have no direct association with COVID-19 (Tables 3 and 4). Several previous studies have reported similar findings. The proportions of CRD among inpatients with COVID-19 range from 25-50% in previous studies ^(43), (44), (45)^. However, the definition of CRD has not been globally established and is inconsistent between studies. That is, there is a remarkable gray zone in determining CRD, which may be having a significant impact on national and regional mortality statistics. Owing to the inconsistency in our study between the cause of death categorizations using the Japanese public criteria (the MHLW criteria) ^(26)^ and our own system, the simple proportions of death associated with COVID-19 appeared different; however, this difference was not statistically significant (70% vs. 35%, respectively, *p* = 0.35) (Table 5). Notably, our method used the WHO criteria and established causality through careful review of the medical records and death certificates, whereas the Japanese public criteria use only death certificate data. The conclusion that the most common causes of death in older inpatients with COVID-19 are non-COVID-19-related may vary depending on the classification system used.

Long COVID has been defined as “an infection-associated chronic condition that occurs after SARS-CoV-2 infection and is present for at least 3 months as a continuous, relapsing and remitting, or progressive disease state that affects one or more organ systems” ^(46)^. The risk of long COVID developing can be reduced by vaccination, antiviral therapy during the acute phase of COVID-19 ^(47), (48), (49), (50)^, and some other types of medications such as antioxidants, antidiabetics, and immunomodulators ^(51)^. In contrast, the post-COVID-19 complications in our study might have been caused by general functional reduction, which often occurs after infectious diseases in older adults ^(52), (53), (54), (55)^. However, they may also have resulted from the same etiology as long COVID. This possibility was suggested by decreased eating function being more common and more severe in the death group, in whom COVID-19 antiviral use was less prevalent (Table 2). Nevertheless, long COVID was unlikely in most patients in the death group, because death occurred within 3 months of COVID-19 onset (Figure 3). These discussions regarding potential therapeutic implications are speculative and require further investigation.

It should be noted that the participants in this study were limited to patients with mild-to-moderate COVID-19. However, in the Omicron-variant era, such patients are less likely to be hospitalized ^(56)^. Previous cohort studies on COVID-19 in patient outcomes have primarily used data that included younger participants from large-scale educational hospitals with abundant medical resources ^(41), (43), (49), (50), (51)^. In contrast, most patients in primary care rural hospitals are older than 65 years, and these hospitals cannot accept patients with severe COVID-19 owing to a lack of intensive care resources.

### Limitations

This study had some limitations. First, it was a single-center study with a relatively small sample. Moreover, multivariate analysis could not be performed to identify mortality risk factors. More large-scale cohort studies are needed to obtain more reliable evidence of the outcomes of older inpatients with COVID-19 in this Omicron-variant era. Second, no data were available on which SARS-CoV-2 variant the participants were infected with. However, surveillance data of the SARS-CoV-2 genome lineage in our area have revealed that almost all patients with COVID-19 during the study period had the Omicron variant ^(25), (38)^. Third, owing to the limited resources available in isolation wards, some of the diagnoses of complications and causes of death in the medical records and on death certificates may have been inaccurate. The patients in isolation could not undergo many important examinations, such as imaging, endoscopy, and invasive diagnostic tests. Fourth, data on patient vaccination histories were incomplete. The attending doctors did not collect this information if it was not needed for treatment planning. Nevertheless, the patient data used in this study were more comprehensive and clinically informative than those used in previous studies to determine the causes of death in this population.

In conclusion, despite only mild-to-moderate severity of COVID-19 among our cohort, we found a relatively high mortality rate among older inpatients. Importantly, the cause of death in most patients was not COVID-19 but unrelated complications and pre-existing medical conditions. Our results indicate that clinicians must pay the utmost attention to preventing complications and controlling comorbidities in older inpatients with COVID-19. Although more large-scale cohort studies are warranted to clarify survival outcomes in this patient population, we hope that these preliminary results will be useful to health care providers working with hospitalized, older patients with COVID-19 in primary care settings.

## Article Information

### Acknowledgments

The authors thank all members of the COVID-19 Working Group of Nanto Municipal Hospital (Makino Okamoto, Syougo Ushikoshi, Keiko Kaji, Aya Nakada, Takaaki Nishimura, Michihiro Noda, Ayane Hasegawa, Natsuki Matsunaga, Naomi Yamada, Shinji Yoshioka, Masahisa Arahata, and Maine Takemura) for their excellent work and efforts.

### Author Contributions

Masahisa Arahata mainly designed and conceived this study. Maine Takemura and Masahisa Arahata contributed to the acquisition, analysis, and interpretation of the data. Masato Kuriyama partially contributed to the work process. Maine Takemura drafted the manuscript. All authors critically reviewed and substantially revised the manuscript. All authors approved the final version of the manuscript and agreed to be accountable for all aspects of the work.

### Conflicts of Interest

None

### IRB Approval Code

Shiminbyouin-932

Name of the Institution: Institutional review board (IRB) of Nanto Municipal Hospital.
